# Reward and Imitation in Social Conformity

**DOI:** 10.1002/brb3.71607

**Published:** 2026-07-16

**Authors:** Garrett Mauter, Mimi Liljeholm

**Affiliations:** ^1^ Department of Cognitive Sciences University of California Irvine California USA; ^2^ Center for the Neurobiology of Learning & Memory University of California Irvine California USA; ^3^ Irvine Center for Addiction Neuroscience University of California Irvine California USA

**Keywords:** imitation, observational learning, reinforcement learning, social conformity

## Abstract

**Purpose:**

Normative social conformity has been proposed to elicit a hedonic reward signal that is dissociable from informational inferences about decision outcomes. If present, such a signal should reinforce not just the decision that preceded it but also incidental, contextual, and stimulus features. Here, we pit this account against a non‐hedonic imitation algorithm.

**Method:**

Across two studies (*n* = 359), we used a nondeceptive three‐participant gambling task in which trial‐by‐trial information was provided about the selections and monetary payoffs of two other participants facing the same, recurring, options in real time. We use computational cognitive modeling to assess the relative contributions of monetary reward, social reward, and non‐hedonic imitation to norm alignment.

**Findings:**

Consistent with both social reward and imitation accounts, but contrary to mere monetary maximization, the probability of staying with a losing option increased with the degree of decision unanimity. However, contrary to the social reward hypothesis, only monetary payoffs modulated the valence of incidental gambling stimuli. A hybrid weighted sum of the two models failed to improve on imitation performance, and a prosocial framing did not significantly alter the pattern of results.

**Conclusions:**

Our findings suggest a prominent role for non‐valanced imitative processes in normative conformity. Future work will address how social and informational constraints shape the roles of imitation and reward in social conformity and observational learning.

## Graphical abstract

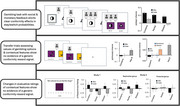

## 1 Introduction

In human societies, access to essential resources often depends on social inclusion. It is not strange, therefore, that humans tend to align their judgments with apparent group norms, ensuring survival contingent on group affiliation. Still, relatively little is known about the exact mechanisms by which norm alignment persists in the absence of concrete informational or economic gain, as when anonymously judging the attractiveness of a face or the palatability of a food (Klucharev et al. 2009; Campbell‐Meiklejohn et al. 2010; Zaki et al. 2011; Nook and Zaki 2015). One common claim is that normative conformity elicits the same hedonic reward signal as that triggered by conventional rewards, such as food or money, together with corollary reinforcement learning (Klucharev et al. 2009; Campbell‐Meiklejohn et al. 2010; Zaki et al. 2011; Nook and Zaki 2015; Izuma 2013, 2017; Wake and Izuma 2017; Messimeris et al. 2023; O'Doherty et al. 2003; Mistry and Liljeholm 2018; Ruff and Fehr 2014). Alternatively, normative conformity might reflect a valence‐neutral action‐copying algorithm, previously considered a basis for imitative observational learning (Najar et al. 2020; Over and Carpenter 2012; Acerbi et al. 2011; Charpentier and O'Doherty 2018; Charpentier et al. 2020; Collette et al. 2017). Though not mutually exclusive, social reward and imitation accounts have different implications for the modulation of conformity behavior by motivational and affective states (Arroyo and Liljeholm 2024) and for the flexibility of conformity‐induced decisions (Liljeholm et al. 2012). Our goal was to characterize the relative contributions of reward and imitation to normative conformity in motivated behavior.

**FIGURE 1:**
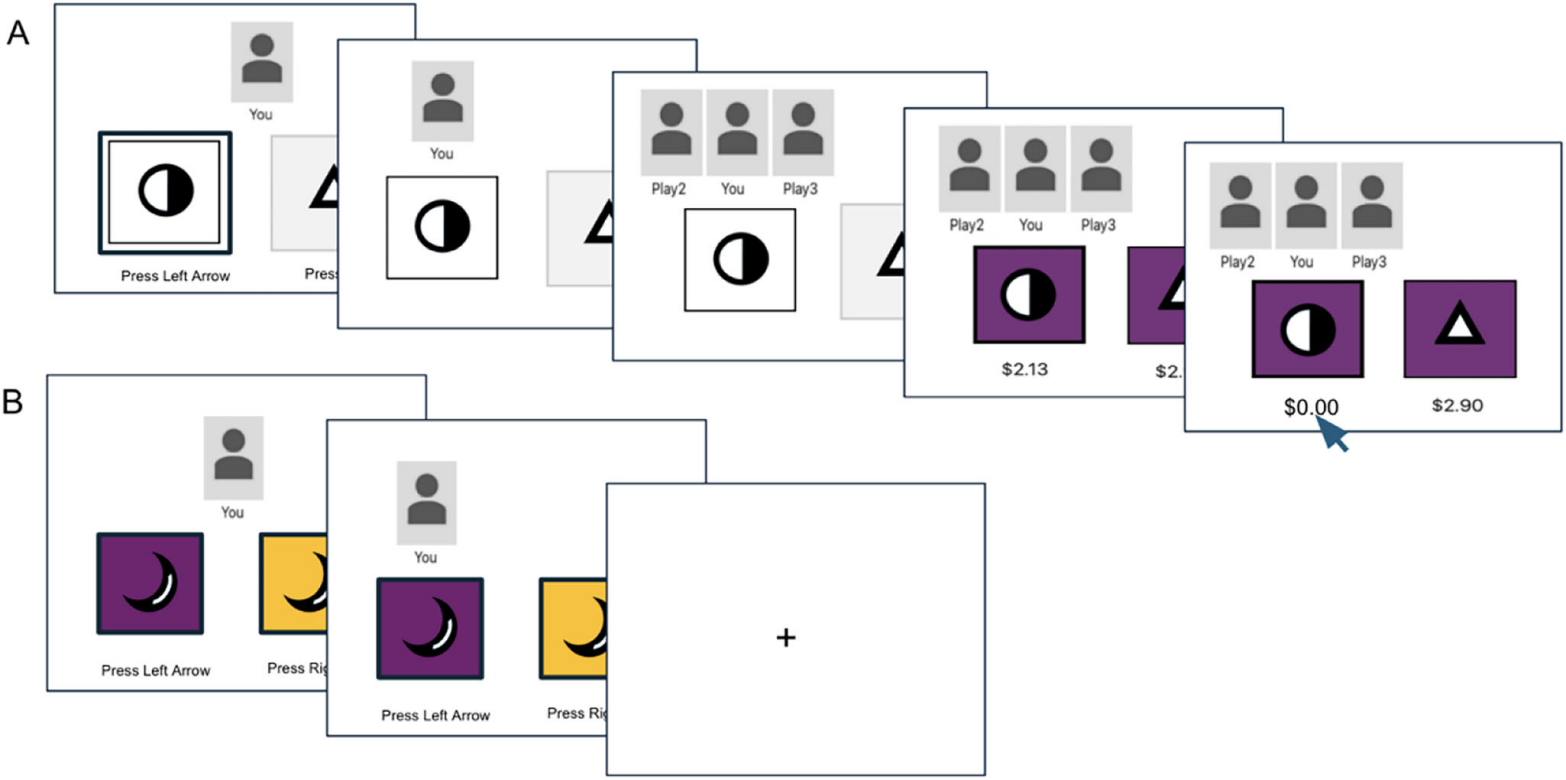
Trial illustration. (A) On gambling trials, participants are presented with two gambling options (abstract black and white shapes). Upon selection, the participant's avatar, initially positioned top center, moves to align with the chosen option. On the subsequent feedback screen, the decisions of the other two participants are likewise indicated by the alignment of their avatars, followed by the display of monetary payoffs beneath each option and a color change applied to both options, with each color corresponding to one of six outcome scenarios (see text). Finally, the lower of the two payoffs is set to zero (arrow) to emphasize the monetary difference between trial outcomes. (B) On transfer trials, different‐color options with novel shapes are presented, but no social or monetary feedback is provided following selection.

To evaluate the claim that normative conformity elicits a generic reward signal, we integrated social and conventional (monetary) rewards in a multi‐participant gambling task, administered online, with individuals participating in groups of three. On each trial, all participants in a group were presented with a pair of abstract shapes (drawn from a set of six), prompted to select one of the two, and following selection, shown the monetary payoffs of both options on that trial, as well as the selections made by the other two participants (see Figure 1A). To further emphasize the monetary difference between trial outcomes, the lower of the two payoffs was reduced to zero following the initial monetary feedback. Thus, six outcome scenarios of interest were generated by combining levels of social alignment (i.e., with one, both, or neither of the other participants) with a monetary gain versus opportunity cost. To ensure variability in decision unanimity, both options presented on a given feedback trial were drawn randomly from the *same* reward distribution and modified to ensure at least a $0.1 difference. Although this generated necessary uncertainty about the “correct” choice on a given trial, the trial‐based feedback categorically ruled out the validity of other participant's decisions as reliable sources of information about decision outcomes.

A fundamental yet untested prediction of the social reward account, distinguishing it from the imitation account, is that *contextual* stimulus features, unrelated to instrumental decisions, should absorb hedonic reward signals triggered by conformity decisions. To test this prediction, in addition to social and monetary feedback, a condition‐specific contextual background color was applied to *both* options on feedback screens, with a unique color, orthogonal to gambling options and decisions, being repeatedly paired with particular social and monetary outcome scenarios. To assess the transfer of a generic reward signal, elicited by either monetary gain or social alignment, evaluative judgments of color stimuli were obtained before and after the gambling task, and intermittently occurring transfer trials (see Figure 1B) solicited a choice between color options that had been paired with consensus versus dissent, respectively, on preceding trials. Critically, to preclude learning, no feedback about monetary or social trial outcomes was provided on transfer trials, some of which also solicited selection between gambling (i.e., shape) options associated with different reward distributions.

We specified three learning algorithms that differed with respect to their treatment of other's decisions and assessed the relative fit of these models to choice behavior: A baseline model that only considers monetary outcomes as rewarding; a social learner that treats consensus as a surrogate reward; and an imitation model that only considers money as reward but that strives to copy observed decisions in parallel with reward maximization. Both the social reward and imitation account predict a tendency to repeat observed decisions, even in the face of a monetary loss. We assessed the probability of staying with an option that had incurred a loss on a previous trial when that decision had involved consensus versus dissent. Finally, although all models predict a transfer of value to contextual stimuli that covary with monetary gain, only the social‐reward learner predicts that such value transfer, assessed here using choice preferences and changes in evaluative ratings, will also occur based on majority alignment.

## 2 Study 1

### 2.1 Methods

#### 2.1.1 Participants

A total of 120 participants (58 females, mean age = 33.2 ± 10.9, age range = 19–68; 63.4% White, 15.1% Black, 10% Asian, 6.7% mixed, 4.2% other) were recruited and completed the study on Prolific (www.prolific.com) for monetary payment, including a base payment of $10 and a performance‐based bonus, ranging from $0 to $10. All participants were based in the United States. One participant was excluded for pressing the left key on 97% of the trials, exceeding a predetermined exclusion threshold of 90% same‐key responses, indicating a failure to actively engage in the task. All participants gave informed consent, and the Institutional Review Board at the University of California, Irvine, approved the study. All aspects of the study conformed to the guidelines of the 2013 WMA Declaration of Helsinki. The sample size was set to match a methodologically similar multiplayer task manipulating social information and objective performance (Asch 1951).

#### 2.1.2 Task and Procedure

Upon joining the study and providing consent, participants were asked to wait until the group totaled three individuals (average wait time was ∼3 min). To enhance the individuality of players, each participant was asked at the start of the study to enter a unique “player name,” consisting of a 1–10‐character string, to be displayed beneath their icon. They were instructed that they would be selecting between various gambles with different monetary payoffs, individually but in parallel, and that, on each gambling trial, they would receive feedback about each other's decisions and monetary outcomes as well as their own. On each trial, all participants made their selections independently (within 10 s or the trial was canceled), after which all choices were simultaneously revealed to all three participants. Before the gambling phase, participants were asked to rate the pleasantness of each of six distinct colors, to be used as contextual stimuli, on a scale from 0 (not at all pleasant) to 10 (extremely pleasant).

Critically, the two (of six) distinct shapes appearing as options on each gambling trial had been randomly assigned at the start of the experiment to *the same* two reward distributions with “high” (*μ* = $4.5 ± $1.5) and “low” (*μ* = $2.0 ± $1.5) means, respectively. Once all participants had made their selection, monetary payoffs for both options were drawn from the shared distribution, one of which was modified if needed to ensure at least a $0.1 difference. The payoffs were then displayed on the screen, together with the group alignment, and the lower of the two was set to $0 in order to emphasize the win versus loss outcome of the trial. One shape from each distribution was included to provide variability in order to prevent fatigue and disengagement and thus occurred with about half the frequency of the other four shapes. At the end of the study, three trials were randomly drawn from all feedback trials, and each participant received the sum of their individual earnings on those three trials, ranging from $0 to $10.

The group alignment on each gambling trial (see Figure 1A) occurred naturally as a consequence of participants’ choices, without experimental deception or manipulation, and independently of monetary outcomes, since both options were associated with the same monetary reward distribution. In contrast, a unique outcome color was applied to *both options* following choice that depended on the particular combination of monetary and social outcomes. According to the social reward hypothesis, these contextual background colors should absorb reward signals generated by decision unanimity as well as monetary reward. Four unique colors were counterbalanced across the four critical conditions: (1) win (selected the greater payoff option) and full consensus (both of the other two participants selected the same option), (2) win and dissent (neither of the other two participants selected the same option), (3) lose (selected the lesser payoff option, resulting in a $0 trial outcome) and full, and (4) lose and dissent. To reduce the color counterbalancing load, two separate colors were counterbalanced across partial‐win and partial‐lose conditions, for which predictions are somewhat equivocal and which necessarily occur about twice as often as full consensus and dissent trials.

The acquisition of valence by background colors was assessed using evaluative ratings and choice preferences. First, participants were asked to rate the pleasantness of each of six distinct colors, to be used as contextual stimuli, on a scale from 0 (not at all pleasant) to 10 (extremely pleasant), both before and after gambling. Ratings obtained prior to gambling were subtracted from ratings obtained following gambling to compute a measure of change in color affect. Second, intermittent “transfer” test trials required a selection between options with identical novel shapes but with different background colors, each respectively paired with consensus versus dissent, and both associated with either win or loss outcomes on gambling trials (see Figure 1B). Critically, no social or monetary feedback was provided on these trials to prevent learning effects. To confirm that participants were tracking monetary payoffs, additional transfer trials forced selection between shape options associated with high versus low monetary payoffs, again without providing feedback. There were a total of 150 trials, with 30 transfer trials (10 per comparison) interspersed among 120 gambling trials.

In addition to the gambling task and pleasantness ratings, participants completed surveys assessing individual differences in individualism and collectivism (Triandis and Gelfand 1998), narcissism (Gentile et al. 2013), and social anxiety (Łakuta 2018). These questionnaires were administered at the end of the experiment for independent data mining purposes and are noted here only for completeness but are available in our open‐source materials. To ameliorate completion load, these were administered between participants, with each participant receiving either the individualism and collectivism surveys or the narcissism and social anxiety surveys, in counterbalanced order.

#### 2.1.3 Computational Modeling and Statistical Analyses

We specified three error‐driven learning rules that differed with respect to their treatment of other's decisions and assessed the relative fit of those models to choice behavior. First, a baseline model considered only monetary outcomes as rewarding:

(1) $$V\left(s\right)\leftarrow V\left(s\right)\alpha \left[\$\left(s^{\prime }\right)-V{\left(s\right)}_{\$}\right]$$

where *V*(*s*) is the value estimate for stimulus, *s* (i.e., a gambling option or background color), α is a learning rate parameter and $(*s′*) is the monetary payoff paired with option *s*. Second, a social reward learner that treats majority alignment as a surrogate reward that scales with majority size:

(2) $$V\left(s\right)\leftarrow V\left(s\right)+\alpha \left[\$\left(s^{\prime }\right)+\gamma c\left(s^{\prime }\right)-V\left(s\right)\right]$$

where *c*(*s′*) is the conformity outcome, reflecting alignment with 2, 1, or 0 other players, and *γ* is a free parameter estimating the subjective utility of conformity. Finally, we specify an *imitation* model that treats only monetary outcomes as rewarding but that strives to copy observed decisions in conjunction with reward maximization:

(3) $$V\left(s\right)\leftarrow V\left(s\right)+\alpha \left[\$\left(s^{\prime }\right)-V{\left(s\right)}_{\$}]+\beta [c\left(s^{\prime }\right)-V{\left(s\right)}_{C}\right]$$

where β is a learning rate parameter for action copying. A hybrid of the social reward and imitation models (i.e., a model that sums the *β*− and *γ*‐weighted terms in Equations 2 and 3, respectively) was also considered.

Note that, in addition to variations in the frequency of gambling options, the task necessarily generates more partial consensus trials than full consensus or dissent trials. We added a UCB1 term to value estimates to account for different event frequencies in both gambling options and incidental features, and the resulting value was passed to a softmax rule with a free noise parameter, *τ*, that generated decision probabilities. Models were fit to behavioral data by minimizing the negative log likelihoods, and the Akaike information criterion (AIC) was used for model comparisons. Parameter recovery was assessed by simulating 1000 players, each receiving payoffs and social information drawn from randomly selected participant data, repeated across 10 training cycles, with randomly generated parameter values from the range used in model fitting, and with Pearson's correlation coefficients estimating recovery.

We use planned comparisons and repeated measures analyses of variance (ANOVAs) to assess the influence of decision unanimity on behavioral gambling and transfer test trials. We use the term “es” throughout to indicate effect sizes for *t*‐ (Cohen's d) and *F*‐ (partial eta squared) tests. All tests are two‐tailed.

### 2.2 Results

With respect to model performance, mean AIC scores, penalizing for free parameters, were lower, indicating better performance, for the imitation model than for the social reward, *t*(118) = −3.74, *p *< 0.001, *effect size (es)* = −0.34, 95%, CI [−10.07, −3.10] and baseline, *t*(118) = −4.08, *p *< 0.001, *es *= −0.37, 95% CI [−9.90, −3.43], models. A Hybrid model that included both the imitation and social reward terms failed to outperform the imitation model, which again produced a lower mean AIC score, albeit not significantly so, *t*(118) = −0.74, *p *= 0.46, *es = *−0.07, 95% CI [−1.44, 0.66]. The superior performance of the imitation model was also apparent in the number of participants for which the imitation model (89/119) generated a lower AIC score than the hybrid model. Parameter recovery was excellent across parameters and models (*r > *0.93 and *p < *0.001). Mean best‐fitting parameters, listed in the first columns of Table 1, suggested a greater weighing of the imitation parameter across models.

**TABLE 1:** Best fitting parameter values from Study 1 and both groups in Study 2.

		Study 1				Study 2—replication				Study 2—prosocial			
	*α*	*β*	*γ*	*τ*	*α*	*β*	*γ*	*τ*	*α*	*β*	*γ*	*τ*
Hybrid	0.06	0.09	0.07	1.17	0.06	0.15	0.10	1.07	0.05	0.11	0.06	1.09
Imitation	0.05	0.13	—	1.14	0.04	0.17	—	1.06	0.04	0.14	—	1.10
Soc. rew.	0.06	—	0.08	1.11	0.06	—	0.15	1.06	0.05	—	0.08	1.07
Baseline	0.06	—	—	1.10	0.05	—	—	1.05	0.04	—	—	1.06

**FIGURE 2:**
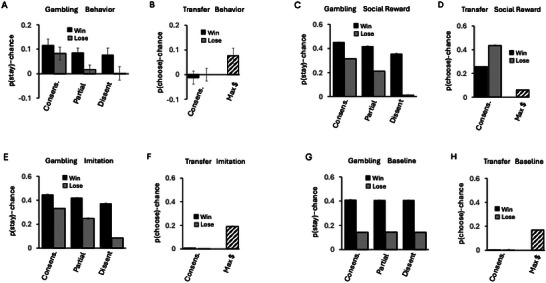
Behavioral and simulated choice proportions with chance performance subtracted, from Study 1. (A) The probability (minus chance) of staying with an option that occurred on the previous trial, as a function of decision unanimity (consensus, partial, and dissent) and monetary (win vs. loss) outcome on that previous trial. (B) Choice proportions (minus chance) on transfer trials without feedback. Left two bars show preference for contextual stimuli (background colors) paired with consensus (over dissent) in win and lose conditions, respectively. Rightmost bar shows degree of preference for gambling options associated with high versus low mean reward distributions. Parts C–D, E‐F, and G‐H  show, respectively, the corresponding gambling and transfer predictions generated by simulated social reward, imitation, and baseline learners. Error bars = SEM.

We operationalized social conformity as the tendency to stay with a gambling option given the unanimity with which it was selected on the previous trial, if repeated (regardless of left/right positions on the screen). Mean behavioral stay probabilities as a function of decision unanimity and monetary payoffs are plotted in Figure 2A, together with corresponding choice proportions from model simulations (Figure 2C,E,G). Our planned comparison (see Section 1) confirmed that, as predicted by both the social and imitation accounts, but not the baseline (monetary) account, the probability of staying with an option that had incurred a *loss* (i.e., $0 payoff) on the previous trial was significantly greater for consensus decisions than for dissent decisions; *t*(110) = 2.30, *p = *0.02, *es *= 0.22, 95% CI = [0.01, 0.15]. A repeated measures ANOVA with consensus and payoff as within‐subject factors revealed significant effects of consensus [*F*(2,214) = 3.50, *p *= 0.03, *es* = 0.03] and payoff [*F*(1,107) = 9.73, *p =* 0.002, *es* = 0.08], but no interaction (*p* = 0.84).

Although both imitation and social reward learners predict an influence of consensus on stay probabilities, the social reward model alone predicts that majority alignment will elicit a generic reinforcement signal that transfers not only to relevant decision variables but also to incidentally occurring contextual features. We assessed such reinforcement using transfer trials, in which colors *differed* across gambling options, one being deterministically associated with full consensus decisions and the other with dissent from both other participants. Mean choice preferences for consensus over dissent colors and for monetary win and loss colors are illustrated on the left side of Figure 2B. Planned comparisons confirmed that there was no significant consensus preference for either win (*p* = 0.68) or loss (*p* = 0.50) colors. In contrast, as shown on the right side of Figure 2B, transfer trials that assessed a preference for gambling options associated with greater monetary payoffs revealed a clear effect; *t*(118) = 3.07, *p* = 0.003, *es* = 0.28, 95% CI = [0.03, 0.12].

**FIGURE 3:**
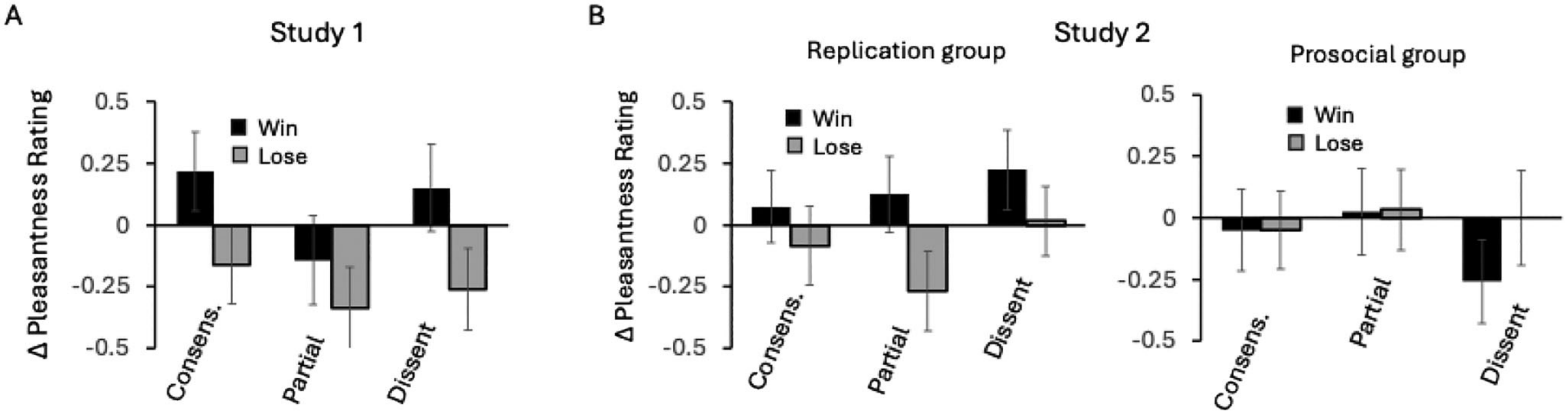
Changes in evaluative judgments. The difference between pleasantness ratings of contextual color stimuli obtained before and after the gambling phase, plotted as a function of their pairing with a particular level of group alignment (full, partial, or none) and with win versus lose monetary outcomes where a loss indicates a $0 trial payoff: (A) results from Study 1 and (B) results from the replication (left) and prosocial (right) groups in Study 2. Error bars = SEM.

Finally, a consensus‐induced change in the valence of condition‐specific background colors was assessed using pleasantness ratings solicited before and after the gambling session. The difference between pre‐and post‐gambling ratings, as a function of social and monetary contingencies, is plotted in Figure 3A. A repeated measures ANOVA performed on the difference between pre‐ and post‐gambling ratings, with consensus and win/lose payoff as factors, revealed a significant main effect of payoff, *F*(1,118) = 5.34, *p =* 0.023, *es* = 0.04, indicating that participants' affect did indeed change for contextual features that coincided with monetary gain, but there was no significant effect of consensus and no interaction (*p *> 0.29), again contrary to the social reward hypothesis.

### 2.3 Interim Discussion

In Study 1, we found clear evidence of normative conformity in that participants were significantly more likely to stay with a losing option that repeated on the subsequent trial if it had been unanimously selected than if both other participants had selected the other alternative. We also found clear evidence of monetary reward maximization and of changes in affect associated with contextual stimuli on the basis of monetary payoffs; however, critically, there was no evidence of changes in contextual affect on the basis of consensus versus dissent. One limitation of these results is that monetary reinforcement of contextual features was assessed using evaluative judgments only, whereas social reinforcement, pivotal for ruling out a social reward surrogate, was assessed using both evaluative judgments and transfer trials during gambling. We address this limitation in Study 2.

Another potential weakness of Study 1 is that it may be lacking conditions that are necessary for conformity to become rewarding. Although previous claims about the intrinsic reward of social conformity have not posited any contextual constraints (Klucharev et al. 2009; Baumeister and Leary 1995; Asch 1951; Izuma et al. 2008; Mistry and Liljeholm 2019), a large number of studies have focused on conformity in prosocial contexts (Nook et al. 2016; House 2018; van Baaren et al. 2004; Müller et al. 2012). In Study 2, one group of participants made all gambling decisions knowing that any monetary earnings would be donated to a charity of their choice, rather than benefit themselves financially. We hypothesized that the well‐documented peer pressure mediating prosocial decisions might make majority alignment more rewarding, shifting the evidence towards the “conformity as reward” account.

## 3 Study 2

### 3.1 Methods

#### 3.1.1 Participants

A total of 240 participants were randomly assigned to a replication group (63 females, mean age = 41.7 ± 11.2, age range = 20–74; 71% White, 6.7% Black, 9.2% Asian, 5.8% mixed, 7.5% other) and a prosocial group (51 females, mean age = 41 ± 10.1, range = 19–65; 69.1% White, 7.5% Black, 9.2% Asian, 6.7% mixed, 7.5% other) and completed the study on Prolific (www.prolific.com) for monetary payment, including a base payment of $10 and a performance‐based bonus, ranging from $0 to $10, benefiting either the participant (replication group) or a charity of their choice (charity group). All participants were based in the United States. The sample size was set to match that of Study 1, with no exclusions. All participants gave informed consent, and the Institutional Review Board of the University of California, Irvine, approved the study. All aspects of the study conformed to the guidelines of the 2013 WMA Declaration of Helsinki.

#### 3.1.2 Task and Procedure

The task and procedures were identical to Study 1, except that in one group participants received instructions that all earnings would be donated to a charity; once the gambling phase was completed, participants in this group were prompted to select a charity from a 40‐item list of American and international charities, to which their bonuses were subsequently donated. As in Study 1, exploratory trait‐assessment questionnaires were administered after all task elements had been completed. Contrary to Study 1, background colors assigned to partial consensus conditions were fully counterbalanced across all conditions.

### 3.2 Results

A mixed ANOVA performed on the AIC modeling scores with group (replication vs. prosocial) as between‐subjects factor and model as a within‐subjects factor revealed a significant main effect of model, *F*(3,714) = 42.64, *p *< 0.001, but no significant main effect of group (*p* = 0.91) or interaction (*p* = 0.85). As in Study 1, AIC scores were lower, indicating better performance, for the imitation model than for the social reward, *t*(239) = −7.81, *p *< 0.001, *es =* −0.51, 95% CI [−12.35, −7.38], and baseline, *t*(239) = −7.99, *p <* 0.001, *es *= −0.52, 95% CI [−12.87, −7.78], models. The hybrid model, combining imitation with social and monetary reward, again generated greater AIC scores than the imitation model, this time significantly so, *t*(239) = −2.08, *p* = 0.04, *es *= −0.13, 95% CI [−2.73, −0.07], with 190 of 240 participants having lower AIC scores for the imitation model. As in Study 1, parameter recovery was excellent (*r > *0.93 and *p < *0.001), and mean best‐fitting parameters listed in Table 1 suggested a greater weighing of imitation than social reward.

**FIGURE 4:**
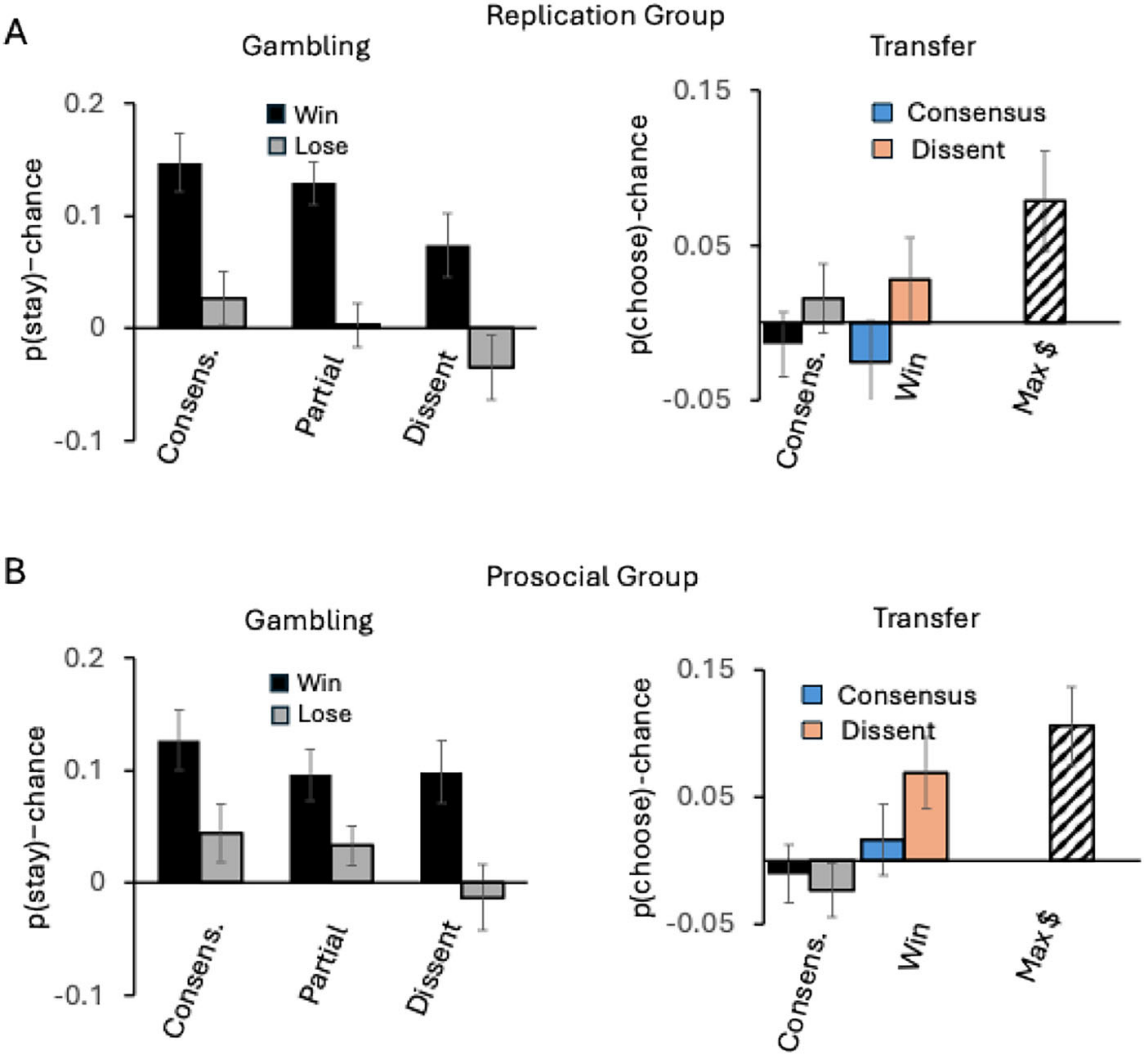
Behavioral results from Study 2. (A) Replication group. Left: the probability (minus chance) of staying with an option that occurred on the previous trial, as a function of decision unanimity (consensus, partial, and dissent) and monetary (win vs. loss) outcome on that previous trial. Right:choice proportions (minus chance) on transfer trials without feedback; the leftmost two bars show preference for colors paired with Consensus over colors paired with dissent, in win and lose conditions, respectively; blue and orange bars show preference for colors paired with monetary Wins over colors paired with $0 payoffs, on Consensus and Dissent trials, repsectively; the rightmost bar shows degree of preference for gambling options associated with high versus low mean reward distributions. (B) Corresponding results in the prosocial group. Error bars = SEM.

As in Study 1, despite apparent conformity in gambling decisions, no significant preference was found for background colors paired with full consensus over those paired with dissent (leftmost two bars in the right panel of Figure 4A,B), in either win (*p =* 0.78) or loss (*p = *0.57) conditions, again suggesting the absence of a generic reward signal elicited by group alignment. In contrast, as shown with the blue and orange bars in the right panel of Figure 4, there was a clear preference for background colors associated with monetary Win (over Loss) outcomes, though interestingly this preference did not emerge for Win colors associated with consensus (*p* = 0.85), only for Win colors associated with dissent; *t*(239) = 2.64, *p* = 0.009, *es* = 0.17, 95% CI = [0.01, 0.09]. As in Study 1, the strongest transfer effects emerged for choices between gambling options drawn from different monetary reward distributions (never pitted against each other on feedback trials). A mixed ANOVA revealed a significant effect of reward distribution [*F*(2,238) = 26.37, *p *< 0.001, *d = *0.10], but no significant effect of group or interaction (*p* > 0.5).

Unlike choice probabilities on feedback and transfer trials, evaluative judgments of contextual cues looked different in the Charity group (right side of Figure 3B) from both the Replication group (left side of Figure 3B) and the Study 1 group (Figure 3A). Specifically, whereas in the replication group, acquired affect again appeared to be solely based on monetary outcomes, those in the charity did not vary consistently with either monetary or social outcomes, resulting in a just‐significant payoff‐by‐group interaction; *F*(1,238) = 3.89, *p *= 0.050, *es = *0.02. No other main effects or interactions were significant (*p > *0.26).

## 4 General Discussion

Across two studies, we assessed the common claim that normative social conformity is mediated by a hedonic reward signal (Klucharev et al. 2009; Campbell‐Meiklejohn et al. 2010; Zaki et al. 2011; Nook and Zaki 2015;Izuma 2013, 2017; Wake and Izuma 2017; Messimeris et al. 2023; O'Doherty et al. 2003; Mistry and Liljeholm 2018; Ruff and Fehr 2014). Specifically, we used a nondeceptive, real‐time, multi‐participant economic choice task to assess whether valence elicited by social alignment transferred to contextual stimuli, according to basic principles of reinforcement learning (Sutton and Barto 1992; Watkins and Dayan 1992). We compared the hedonic reward account with a non‐hedonic action‐copy algorithm, previously argued to support imitative observational learning (Charpentier et al. 2020). Despite clear evidence of normative conformity in economic decisions, only monetary reinforcement, but *not* group alignment, produced changes in the valence of contextual stimuli, consistent with the imitative, but not the social reward, account. In Study 2, a framing in terms of pro‐social decision‐making, which is strongly susceptible to social pressure (Nook et al. 2016; House 2018; van Baaren et al. 2004; Müller et al. 2012; Duell et al. 2021), failed to significantly alter this pattern of results.

Of course, these findings do not categorically rule out a role of social reward in normative conformity, and indeed some aspects of our results did reflect an integration of social and monetary variables, though not one that was well described by a simple weighted sum of social reward and imitative adjustments. Instead, we found that the preference for contextual stimuli associated with monetary wins was specific to dissent outcomes. This asymmetry might reflect a tacit assumption on the part of participants that consensus decisions entailed splitting the payoff across group members and thus that such trials were *less* valuable in terms of monetary profit. Although the existence of such a bias could overshadow an intrinsic utility of consensus on win trials, a clear preference should still have been apparent on loss trials. Moreover, even a shared profit on win trials should result in a preference relative to the loss ($0) outcome, particularly for payoffs drawn from the relatively high reward distribution. We conjecture instead that unanimous decisions may have detracted from the intrinsic utility of “being right,” a significant motivational factor in its own right, not explicitly addressed here. Future work is needed to arbitrate between these possibilities and to better characterize the relative contributions of reward and imitation to normative conformity.

Another important consideration is how the current task fits into the broader conformity literature. As with virtually all studies advancing the conformity‐as‐reward account (Klucharev et al. 2009; Campbell‐Meiklejohn et al. 2010; Zaki et al. 2011; Nook and Zaki 2015; Mistry and Liljeholm 2019; Greenberg and Liljeholm 2021), our study restricted social interactions to the computer screen, with participants making initial judgments that could be revised (e.g., adjusted or switched from) following a subsequently revealed social norm. This is in stark contrast to Asch's seminal line‐judging studies, where in‐person confederate researchers acted as peers whose, objectively incorrect, decisions were presented to the participant prior to their own choice. It may be argued that our approach removes the social pressure necessary for group alignment to elicit a hedonic reward signal. We counter that computer‐based interactions with numerically or graphically represented norms were used in the vast majority of studies advancing the social reward account (Klucharev et al. 2009; Campbell‐Meiklejohn et al. 2010; Zaki et al. 2011; Nook and Zaki 2015; Mistry and Liljeholm 2019; Greenberg and Liljeholm 2021). More importantly, as in those previous studies, we do in fact observe norm alignment, *despite* the lack of in‐person pressure, and even in the face of a monetary opportunity cost. This of course does not rule out an important contribution of hedonic and motivational process to normative conformity in situations where it can be inferred to prevent or reduce negative social experiences, as when facing ostracism or harassment.

We do deviate significantly from previous work on social conformity in two important ways. First, we provided immediate feedback about the trial‐level accuracy of others’ decisions in terms of contingent monetary payoffs, a feature that eliminates any rational use of information‐seeking conformity (since all participants were naïve and the payoffs on gambling trials were drawn from the same distribution) and that reflects real‐world conditions in which exposure to decision‐contingent outcomes undoubtedly shape conformity behavior. Second, we did not employ any deception: Rather than fixing the norm according to pre‐defined experimental conditions (which may lead to hypothesis guessing in extreme cases, as when a group of peers repeatedly and unanimously contradict visual reality), we allow group alignment to evolve dynamically in a real‐time decision environment. We believe that the natural variance in the latencies of others’ responses constitutes an important ecological variable that can be contrasted with artificial response latencies in future work.

The argument that social conformity is driven by a hedonic reward signal has been largely based on demonstrations that the alignment of an individual's subjective judgments (e.g., about the attractiveness of a face) with group norms correlates with BOLD activity in the ventral striatum (VS) (Klucharev et al. 2009; Campbell‐Meiklejohn et al. 2010; Zaki et al. 2011; Nook and Zaki 2015)—an area heavily implicated in reward signaling (O'Doherty et al. 2003; Knutson et al. 2001; McClure et al. 2004; Hare et al. 2009, 2011; Plassmann et al. 2008). However, the VS responds to a range of stimulus dimensions, including aversiveness and novelty (Delgado et al. 2008; Jensen et al. 2003; Robinson et al. 2013; Guitart‐Masip et al. 2010; Del Giacco et al. 2022; Büchel et al. 2017; Wittmann et al. 2008; Schultz 1998), so its mere involvement does not implicate reward processes. Indeed, BOLD activity in the VS has been shown to scale with imitation of simple body movements (Losin et al. 2012) and with timing in vocal imitation (Belyk and Kotz 2025), consistent with the behavioral results presented here. Moreover, subsequent studies, aimed at identifying a common neural signal for social and conventional rewards, failed to do so (Levorsen et al. 2021). This highlights the imperative of high‐validity behavioral experimentation over neural readouts for advancing the understanding of complex psychological processes.

Given their substantial behavioral and neural overlap, one might question the significance of a distinction between reward and imitation in conformity. One important aspect is that reward sensitivity is dysregulated in individuals across a wide range of psychopathologies (e.g., depression, psychopathy, and anxiety) (Cardoso Melo et al. 2023); this work suggests that compensatory imitative mechanisms might preserve some degree of norm alignment in these populations. Beyond individual differences, the inherent reliance of imitative processes on arbitrary stimulus‐response mappings may facilitate the development of inflexible instrumental habits (Liljeholm et al. 2012). In addition to following up on these implications, further work is needed to address limitations of the current paradigm, including the lack of in‐person contact, small group size, and absence of punishing decision outcomes (e.g., large monetary losses); all factors that shape the stakes of gambling decisions and with that perhaps the relative weighting of social reward in conformity behavior.

## Author Contributions

**Garrett Mauter**: investigation, methodology, writing – original draft, formal analysis, visualization. **Mimi Liljeholm**: conceptualization, methodology, investigation, funding acquisition, writing – original draft, visualization, formal analysis, supervision, writing – review and editing.

## Funding

This research was supported by the National Science Foundation Grant 1844632 (M.L.).

## Disclosure

No aspects of the study were preregistered.

## Ethics Statement

All participants gave informed consent, and the Institutional Review Board at the University of California Irvine approved the study. All aspects of the study conformed to the guidelines of the 2013 WMA Declaration of Helsinki.

## Conflicts of Interest

The authors declare no conflicts of interest.

## Data Availability

All study materials, primary data, modeling, and analysis scripts are publicly available at https://osf.io/sn2we/overview?view_only=5b3b9f02f2574dad9b202b4e924342b8.
